# Active immunization to tumor necrosis factor-α is effective in treating chronic established inflammatory disease: a long-term study in a transgenic model of arthritis

**DOI:** 10.1186/ar2897

**Published:** 2009-12-23

**Authors:** Laure Delavallée, Luca Semerano, Eric Assier, Géraldine Vogel, Grégoire Vuagniaux, Marion Laborie, Daniel Zagury, Natacha Bessis, Marie-Christophe Boissier

**Affiliations:** 1 EA4222, Li2P, University of Paris 13, 74 rue Marcel Cachin, 93000, Bobigny, France; 2 Rheumatology Department, Hôpital Avicenne, Assistance Publique-Hôpitaux de Paris (AP-HP), 125 rue de Stalingrad, 93000, Bobigny, France; 3 Neovacs SA, 3-4 impasse Reille, 75014, Paris, France; 4 Debiopharm SA, Chemin Messidor 5-7, Case Postale 5911, CH-1002, Lausanne, Switzerland

## Abstract

**Introduction:**

Passive blockade of tumor necrosis factor-alpha (TNF-α) has demonstrated high therapeutic efficiency in chronic inflammatory diseases, such as rheumatoid arthritis, although some concerns remain such as occurrence of resistance and high cost. These limitations prompted investigations of an alternative strategy to target TNF-α. This study sought to demonstrate a long-lasting therapeutic effect on established arthritis of an active immunotherapy to human (h) TNF-α and to evaluate the long-term consequences of an endogenous anti-TNF-α response.

**Methods:**

hTNF-α transgenic mice, which spontaneously develop arthritides from 8 weeks of age, were immunized with a heterocomplex (TNF kinoid, or TNF-K) composed of hTNF-α and keyhole limpet hemocyanin after disease onset. We evaluated arthritides by clinical and histological assessment, and titers of neutralizing anti-hTNF-α antibody by enzyme-linked immunosorbent assay and L929 assay.

**Results:**

Arthritides were dramatically improved compared to control mice at week 27. TNF-K-treated mice exhibited high levels of neutralizing anti-hTNF-α antibodies. Between weeks 27 and 45, all immunized mice exhibited symptoms of clinical deterioration and a parallel decrease in anti-hTNF-α neutralizing antibodies. A maintenance dose of TNF-K reversed the clinical deterioration and increased the anti-hTNF-α antibody titer. At 45 weeks, TNF-K long-term efficacy was confirmed by low clinical and mild histological scores for the TNF-K-treated mice. Injections of unmodified hTNF-α did not induce a recall response to hTNF-α in TNF-K immunized mice.

**Conclusions:**

Anti-TNF-α immunotherapy with TNF-K has a sustained but reversible therapeutic efficacy in an established disease model, supporting the potential suitability of this approach in treating human disease.

## Introduction

Rheumatoid arthritis (RA) is a chronic autoimmune disease with an estimated prevalence of about 0.5% in the adult population. This disease, characterized by synovial membrane hyperplasia and immune cell infiltration, affects multiple peripheral joints and leads to destruction of bone and cartilage, inducing pain and disability. Although its precise etiology is still unknown, the pro-inflammatory cytokines, such as tumor necrosis factor-alpha (TNF-α), interleukin (IL)-1β, IL-17, and more recently IL-23, have been shown to be critical mediators in the inflammatory process [1]. It has also been demonstrated that TNF-α mediates a wide variety of effector functions in RA, including the release of pro-inflammatory cytokines and chemokines, leukocyte accumulation, angiogenesis, and the activation of endothelial cells, chondrocytes, and osteoclasts [2,3]. Based on the pivotal role of TNF-α in the pathogenesis of RA [4], two classes of biologic drugs to block this cytokine have been developed: a soluble TNF-α receptor (etanercept) and TNF-binding monoclonal antibodies (mAbs) such as infliximab, adalimumab, golimumab, or certolizumab [5,6]. Although they show a rapid and substantial therapeutic benefit in most patients, with a good safety profile, primary unresponsiveness and secondary escape phenomena are not uncommon [7]. Nonetheless, the tremendous success of TNF-α blockade by mAbs has sparked interest in developing alternative strategies for antagonizing TNF-α, such as gene therapy by electrotransfer [8], short interfering RNA [9], or active anti-TNF-α immunotherapy [10-13].

Active immunotherapy is based on the established principles of vaccination. The aim of such a strategy is to use immunization with a protein compound to generate high titers of neutralizing antibodies to a given antigen, which can be either a self-protein or an environmental non-infectious agent. Therapeutic immunization has produced promising results in several fields, and in the case of active immunotherapy against cytokines (AIC), the choice of the target cytokine is informed by the long-term experience with mAbs, receptors, or antagonists in inflammatory and autoimmune diseases [2]. Over the last decade, several active anti-TNF-α immunotherapies using mTNF-α derivates as the immunogen have been developed and tested in murine experimental models of RA [10,11,13].

More recently, with the aim of addressing diseases mediated by human TNF-α (hTNF-α), we developed an anti-hTNF-α compound called TNF kinoid (TNF-K), which is composed of biologically inactive but immunogenic hTNF-α conjugated to a carrier, keyhole limpet hemocyanin (KLH). We have tested TNF-K in hTNF-α transgenic (TTg) mice, which overexpress hTNF-α and develop an erosive polyarthritis that shares many features with RA [14,15]. This model is the only relevant model since anti-TNF antibodies generated by TNF-K target hTNF-α. Previously, we have shown that a prophylactic anti-hTNF-α immunization protected TTg mice OK from developing arthritis [12,16]. To determine the potency of this compound against established arthritis, we immunized TTg mice after the onset of arthritis. We studied the animals for a long time period to evaluate the duration of the potential disease-modulating activity of TNF-K. We showed that TNF-K immunization is efficacious against established arthritis and induces a transient TNF blockade with reversible effects on arthritis in TTg mice.

## Materials and methods

### Animals

Six- to nine-week-old male hemizygous TTg mice (1006-T) were purchased from Taconic Farms (Germantown, NY, USA) [14]. These mice are similar to Tg197 mice and develop a spontaneous arthritis at from 8 to 10 weeks of age [15]. All procedures were approved by the Animal Care and Use Committee of the University of Paris 13.

### Reagents

We obtained hTNF-α kinoid (TNF-K), a protein complex of hTNF-α and KLH, as previously described [16]. Dulbecco's phosphate-buffered saline (PBS) was purchased from Eurobio (Les Ulis, France). ISA-51 adjuvant was obtained from Seppic (Paris, France).

### Therapeutic and long-term effect of TNF-K active immunization

All treatments were started after the onset of arthritis, when TTg mice reached an average clinical score of 3 out of 12. The experimental protocol was as follows (Additional file 1). The control group consisted of eight mice treated with PBS emulsified in ISA-51 adjuvant (PBS group) at 15, 16, and 19 weeks of age. This group was followed for 12 weeks and then euthanized for ethical reasons. A group of 23 TTg mice received three primary intramuscular (IM) injections of TNF-K (4 μg) emulsified in ISA-51 (TNF-K group) at 15, 16, and 19 weeks of age. They were then randomly subdivided into two subgroups of eight and one subgroup of seven TTg mice. The first eight mice were euthanized at 27 weeks of age to compare the TNF-K immunized group with controls. At 32 weeks of age, the subgroup of seven mice received a maintenance dose of TNF-K emulsified in ISA-51 adjuvant, whereas the second subgroup of eight mice received, as a control, an injection of PBS emulsified in ISA-51 at the same time; both were followed until 45 weeks of age. In parallel, another group of eight mice was given weekly intraperitoneal (IP) injections of infliximab (1 mg/kg) from week 15 to week 27. At this time, infliximab was discontinued.

### Antibody assay

From blood samples collected at different time points during the experiment and at sacrifice, sera were obtained and tested for anti-KLH and anti-TNF-α antibody titers and for anti-TNF-α antibody neutralizing capacity. Specific anti-hTNF-α and anti-KLH antibody titers were determined using direct enzyme-linked immunosorbent assay (ELISA) [12]. Precoated ELISA plates with 100 ng per well hTNF-α or KLH were incubated with serial dilutions of sera from immunized and control mice. Specific IgGs were detected by using horseradish peroxidase-conjugated rabbit anti-mouse IgG (Zymed Laboratories Inc., now part of Invitrogen Corporation, Carlsbad, CA, USA). The optical density (OD) was measured at 490 nm for each well.

The neutralizing capacity was assessed by using the L929 cytotoxicity assay, reflecting neutralizing antibodies [12]. Briefly, mouse fibroblast L929 cell line (CCL 1) (American Type Culture Collection, Manassas, VA, USA) was cultured in Dulbecco's modified Eagle's medium containing 10% fetal calf serum. The cells were seeded in flat-bottomed 96-well plates and grown to 95% confluence. After 21 hours of incubation at 37°C, serial dilution of serum with a 100% toxic hTNF-α dose was added on L929 cells with 1 μg/mL of actinomycin D. After 20 hours of incubation at 37°C, the medium was removed and replaced with MTS/PMS during 4 hours at 37°C. The OD at 490 nm was measured for each well. The neutralization titer was expressed as the reciprocal of the serum dilution that neutralizes 50% of hTNFα activity.

### Evaluation of B-memory response after TNF-K immunization

Thirty-six TTg mice received three IM injections of TNF-K emulsified in ISA-51 adjuvant at 7, 8, and 11 weeks of age. They were then randomly subdivided in two subgroups of ten and two subgroups of eight TTg mice. Neutralizing anti-hTNF-α antibody titers were monitored every month. When a decrease of 50% of the neutralizing capacity of these antibodies was observed, mice were intraperitoneally injected with native hTNF-α (10 ng), native hTNF-α (100 ng), KLH (10 μg), or PBS (equivalent volume) 24 weeks after the primary injection. Four weeks later, these mice received IM injections of the same compound with the same doses. The mice were further followed for 10 weeks. The native hTNF-α doses were chosen based on previous results we obtained in a TNF-α-dependent lethal shock experiment, in which we showed that 1 μg of native hTNF-α injections in TTg mice sensibilized with D-galactosamine was enough to kill the mice [12].

### Clinical and histological assessments

Blinded weekly monitoring of body weight and arthritis scores in all four limbs was started from the reception of the animals (9 weeks of age). Clinical severity of arthritis for each paw (fingers, tarsus, and ankle) was quantified by attributing a score ranging from 0 to 3: 0, normal; 1, slight redness and swelling; 2, pronounced edematous swelling of the entire foot; 3, joint deformity and rigidity [12]. The scores of each paw were summed, resulting in an arthritis score ranging from 0 to 12. The mean arthritis score on each clinical observation day was calculated for each treatment group.

For histological assessment of arthritis, all animals were sacrificed after 18-week or 36-week follow-up. Left forelimbs and right hind paws were collected, fixed with formol, decalcified, dehydrated, and included in paraffin blocks. Slides of 5 μm in thickness were made using a microtome. At least four serial sections were realized for each paw in order to obtain a reliable spatial evaluation of articular hints. Slides were then stained with hematoxylin and eosin or with safranin-O before microscopic observation (optical microscope). Synovitis and bone erosions were defined on slides stained with hematoxylin and eosin. Lesions were evaluated quantitatively on each slide using a 3-point scale ranging from 0 to 3, where 0 = normal articulation; 1 = slight inflammation and thickening of the synovium; 2 = mild thickening of the synovium and mild inflammation with invasion of the subsynovial area by inflammatory cells; 3 = severe inflammation and massive invasion of adjacent tissues by pannus [17]. Other sections were scored for loss of safranin-O staining as a measure of cartilage proteoglycan depletion using a scale from 0 to 3, where 0 = no depletion; 1 = depletion of staining and thinning down of the lateral superficial layer; 2 = depletion of staining and thinning down of the central superficial layer; 3 = severe and mostly complete depletion of staining in the superficial layer [18].

### Statistical analysis

Data distribution was preliminarily checked by the Kolmogorov-Smirnov test. Serial measurements of clinical scores, body weight, antibody titers, and antibody neutralizing capacity were analyzed considering the area under the curve for each subject as a summary measure; these measures were then analyzed as raw data [19]. According to data distribution and number of groups, a parametric (analysis of variance [ANOVA], *t *test) or non-parametric (Kruskal-Wallis, Mann-Whitney) test was then performed. *Post hoc *comparisons were performed with the appropriate test according to data distribution (Student-Newman-Keuls for parametric data and Dunn test for non-parametric data). Clinical score time trend was analyzed by Spearman rho, and 95% confidence intervals (CIs) were given. Histological scores were compared with ANOVA or Kruskal-Wallis and their appropriate *post hoc *analysis according to data distribution. Differences in antibody titer at different time points were analyzed with repeated measures ANOVA due to normal distribution of data. Incidences of arthritis were compared using Fisher exact test with Yates correction. All statistics were performed with MedCalc statistical software version 10.4.8 (MedCalc Software bvba, Mariakerke, Belgium).

## Results

### Effect of TNF-K immunization in TTg mice on established arthritis

**Figure 1:**
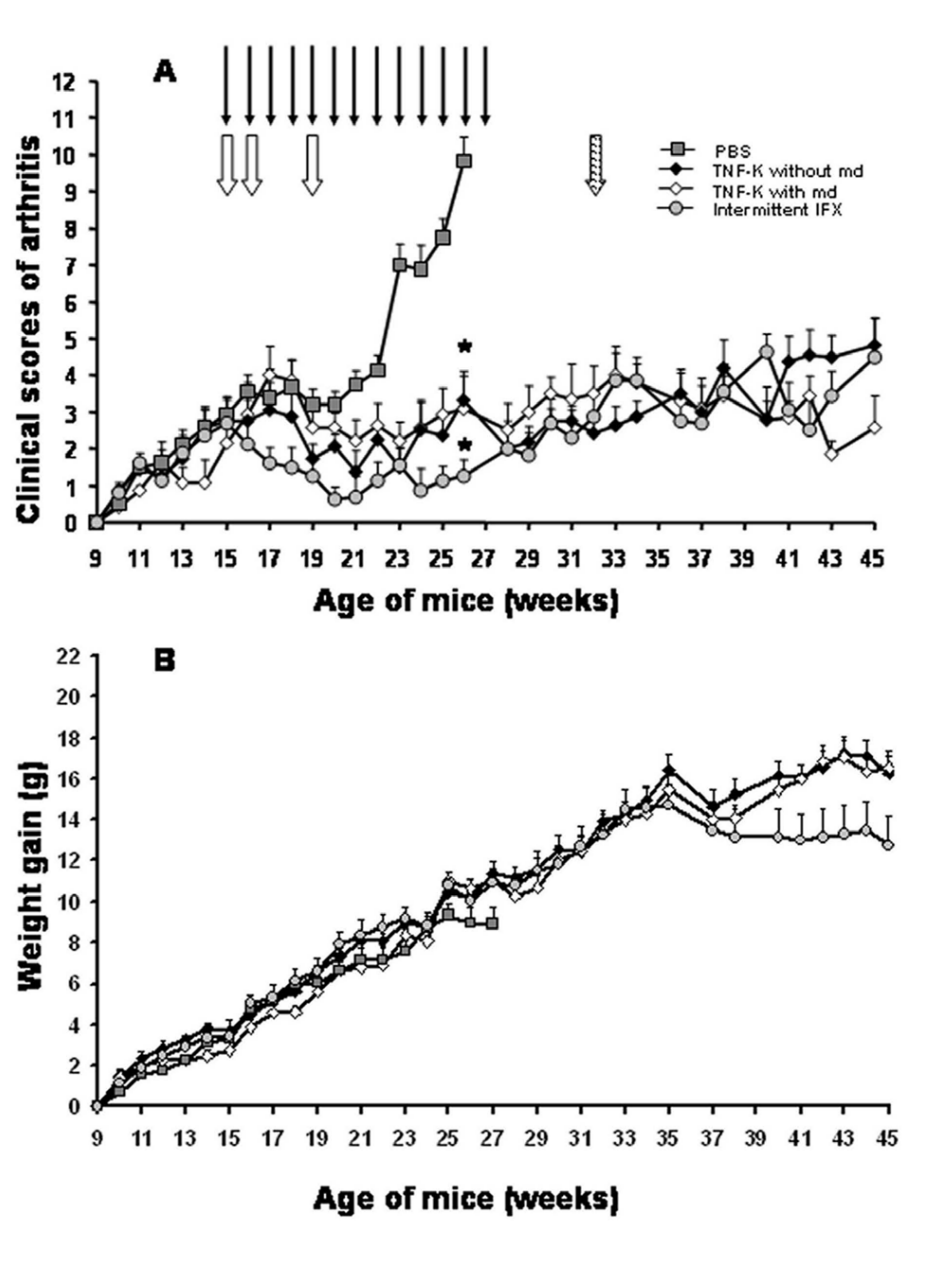
Clinical evaluation of human tumor necrosis factor-alpha transgenic (TTg) mice immunized with tumor necrosis factor kinoid (TNF-K) or phosphate-buffered saline (PBS) or treated with infliximab (IFX). TTg mice were immunized with TNF-K or PBS emulsified in ISA-51 adjuvant or were IFX-treated. All mice were monitored for clinical signs of arthritis and for weight for 18 or 36 weeks. **(a) **TTg mice received three primary injections at 15, 16 and 19 weeks of age (open arrows) of TNF-K (n = 15, open and closed diamonds) or PBS (n = 8, squares). At 32 weeks of age (shaded arrow), TTg mice received a maintenance dose (md) of TNF-K (n = 7, open diamonds) or an injection of PBS emulsified in ISA-51 adjuvant (n = 8, closed diamonds). Eight TTg mice (circles) received weekly intraperitoneal injections of IFX (bold arrows) from week 15 for a period of 12 weeks (until 27 weeks of age). **(b) **The weight gain of all groups is represented. Results are expressed as mean ± standard error of the mean. *P < 0.05 versus PBS.

We investigated the potency of anti-hTNF-α immunization against established arthritis. To address this question, TTg mice, which develop spontaneous arthritis at around 8 to 10 weeks of age, were monitored for any signs of clinical arthritis from 9 weeks of age. When the mice exhibited an average clinical score of 3 (scoring range from 0 to 12; see Materials and methods), treatments were started for all of the mice. The control group (eight mice) was injected with PBS emulsified with ISA-51 adjuvant (PBS group) at 15, 16, and 19 weeks of age and developed severe arthritis over a 12-week period. At 27 weeks of age, these mice were euthanized for ethical reasons (Figure 1a). Compared with the control group, TNF-K immunized mice, receiving injections following the same time schedule, showed a dramatic improvement of the disease after immunization (*P *< 0.05 versus control group) (Figure 1a), demonstrating good efficacy of the TNF-K treatment against established arthritis. TNF-K immunized mice exhibited lower peak clinical scores and fewer inflamed paws than control animals (data not shown). The infliximab-treated group showed, as expected, a significant improvement of the disease (Figure 1a), with lower scores than the PBS group (*P *< 0.05 at week 27). Based on a comparison of clinical scores, the TNF-K immunized and infliximab-treated mice showed comparable efficacy, with no statistically significant differences, although the infliximab has a more rapid efficacy than TNF-K immunization. We did not observe significant differences in body weight in any studied group (Figure 1b).

**Figure 2:**
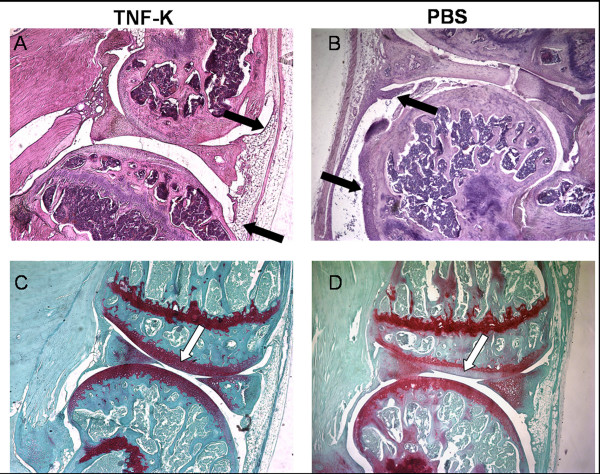
Examples of histological evaluation of tumor necrosis factor-alpha transgenic (TTg) mice immunized with tumor necrosis factor kinoid (TNF-K) or phosphate-buffered saline (PBS). Histological sections (magnification × 40) of the knees of TNF-K- or PBS-treated mice were prepared (see Materials and methods) and colored with hematoxylin and eosin **(a, b) **to observe synovial inflammation or with safranin-O **(c, d) **to observe cartilage degradation. For the histological sections of TTg mice immunized with TNF-K, inflammation (a) and destruction (c) were scored at 0; for the control group, inflammation (b) and destruction (d) were scored at 2. Black arrows show thickness and inflammatory infiltration of synovial membrane in (b) and a normal appearance in (a). White arrows show depletion of proteoglycan (a marker for cartilage destruction) in (d) and a normal full-red staining in (c).

We next investigated the histological efficacy of TNF-K vaccine. At 27 weeks of age, eight TNF-K immunized mice and all control animals were euthanized. We observed that the clinical assessment was corroborated by histological evaluation (Table 1). All control mice exhibited significant histological signs of arthritis, whereas all TNF-K immunized mice showed lower inflammation scores compared with the control group (Table 1 and Figure 2a, b). In regard to joint destruction, TNF-K immunized TTg mice did not exhibit any signs of cartilage damage while the control group showed extensive cartilage destruction (*P *< 0.05) (Table 1). We did not evaluate the histological efficacy of infliximab on TTg mice at 27 weeks of age. For histological arthritis, we observed specific diffusion and pale proteoglycan coloration by safranin-O, reflecting cartilage degradation for control PBS mice in comparison with TNF-K-treated animals (Figure 2c, d).

**Table 1:** Histological evaluation of arthritis in human tumor necrosis factor (TNF)-alpha transgenic mice immunized with TNF kinoid

Group	Number of mice	Inflammation score	Incidence	Destruction score	Incidence
TNF-K (3 injections, sacrifice at week 27)	8	0.1 ± 0.1^a^	2/8^b^	0.0 ± 0.0^a^	1/8^c^

TNF-K (3 injections without maintenance dose, sacrifice at week 45)	8	0.6 ± 0.2^d^	6/8	0.2 ± 0.1^d^	3/8

TNF-K (3 injections with maintenance dose, sacrifice at week 45)	7	0.5 ± 0.1^d^	7/7	0.3 ± 0.1^d^	5/7

Intermittent infliximab (sacrifice at week 45)	8	1.4 ± 0.1	8/8	0.9 ± 0.2	7/8

Phosphate-buffered saline (sacrifice at week 27)	8	1.6 ± 0.1	8/8	0.9 ± 0.2	7/8

The incidence of inflammation/destruction as evaluated by histology is the number of mice with a score of inflammation/destruction of at least 0.25. Results are given as mean ± standard error of the mean. ^a^*P *< 0.05 versus phosphate-buffered saline (PBS); ^b^*P *< 0.01 versus PBS; ^c^*P *< 0.05 versus PBS; ^d^*P *< 0.05 versus infliximab. TNF-K, tumor necrosis factor kinoid.

### Reversibility of TNF-α blockade

**Figure 3:**
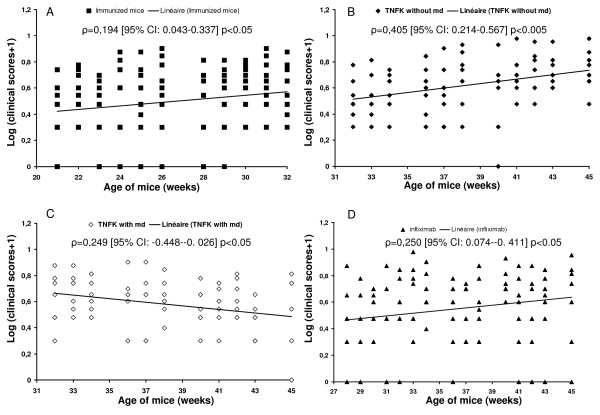
Clinical score time trend. The severity of disease evolution over time was analyzed using Spearman rank correlation. We correlated clinical scores with the age of the mice, expressed in weeks, and divided the study into two periods of time. **(a) **Correlation between week 21 and week 32 for all of the immunized mice (n = 15). We observed an aggravation of disease in all mice immunized with tumor necrosis factor kinoid (TNF-K) a couple of weeks after the last immunization. **(b) **Correlation between week 33 and week 45 for immunized mice not receiving the maintenance dose (md). We observed an aggravation of the severity of the disease. **(c) **Correlation between week 33 and week 45 for immunized mice receiving the maintenance dose. After the maintenance dose at 32 weeks of age, we observed an amelioration of the scores. **(d) **Correlation between week 28 and week 45 for infliximab-treated mice. The injections were stopped at week 27, and we observed an aggravation of the disease over time thereafter. CI, confidence interval.

As TNF-K treatment is able to improve established arthritis based on 12-week follow-up, we investigated the duration of its disease-modulating activity over a longer period. To explore this, we extended by 18 weeks the study of the TNF-K immunized TTg mice for a total study duration of 30 weeks after the first immunization. We observed that, at around 23 weeks of age, arthritis clinical scores started to increase slightly with time (Figure 1a). A time-trend analysis of the clinical scores of both groups having received the primary course of three injections of TNF-K from 21 to 32 weeks of age shows a positive correlation of clinical scores with the age of mice (ρ = 0.194, 95% CI 0.043 to 0.337, *P *< 0.05), demonstrating the transitory effect of anti-hTNF-α immunization (Figure 3a). Furthermore, we observed that, over this period, the number of inflamed paws of TNF-K immunized mice increased compared with that of TNF-K immunized animals sacrificed at 27 weeks of age (*P *< 0.05, data not shown). Histological comparisons were then made between groups of TNF-K immunized mice sacrificed at week 27 and those at week 45. This showed a mild progression of the disease over this 18-week period, with higher inflammation and destruction scores for all of the animals in the week 45 groups (Table 1).

### Effect of a maintenance dose

We next investigated whether this flare in arthritis disease could be ameliorated by the administration of a maintenance dose (late boost) of TNF-K. Therefore, seven TTg mice that had received a primary course of three injections of TNF-K were administered a maintenance dose of TNF-K at 32 weeks of age. As a control, the remaining eight TTg mice that had received the primary course were injected with PBS emulsified in ISA-51 adjuvant. The arthritis clinical score curves decreased for mice that received the maintenance dose and increased for the controls (Figure 1a). The differential in clinical scores between the two groups did not reach statistical significance, and this was due to the small sample size related to effect size. (With an alpha error of 0.05 and a beta error of 0.2, a sample size of 22 mice would have been necessary for the detected difference to be statistically significant.) Nevertheless, clinical score time-trend analysis with Spearman rho showed a reduction of the scores for maintenance-dosed mice (ρ = -0.249, 95% CI -0.448 to -0.026, *P *< 0.05) and a deterioration for controls (ρ = 0.405, 95% CI 0.214 to 0.567, *P *< 0.05), supporting the efficacy of a maintenance dose of TNF-K in treating the late flare of arthritis (Figure 3b, c).

Histological inflammation and destruction were assessed at 45 weeks of age (Table 1). All of the immunized animals exhibited mild signs of histological inflammation and destruction of ankle and knee joints. As with the clinical scores, the differences between immunized animals that received the maintenance dose and those that did not were not statistically significant (Table 1).

We also compared the clinical efficacy of TNF-K active immunization with infliximab intermittent treatment on arthritis of TTg mice over this 18-week extension period. No statistically significant difference was detected between the two treatments (Figure 1a). However, as would be expected, the clinical scores of the infliximab group deteriorated over time since treatment was withdrawn at 27 weeks of age (Figure 3d).

We further examined the histology of infliximab intermittent-treated TTg mice sacrificed at 45 weeks of age. All of the mice from this group, treated with infliximab during 12 weeks, had developed severe inflammation and exhibited mild cartilage destruction of the joints 18 weeks after the infliximab withdrawal (Table 1 and Additional file 1). By comparison, TNF-K immunized animals, receiving or not receiving the maintenance dose, showed lesser inflammation and cartilage destruction compared with the infliximab group (*P *< 0.05) (Table 1).

### Anti-TNFα antibodies after TNF-K immunization

**Figure 4:**
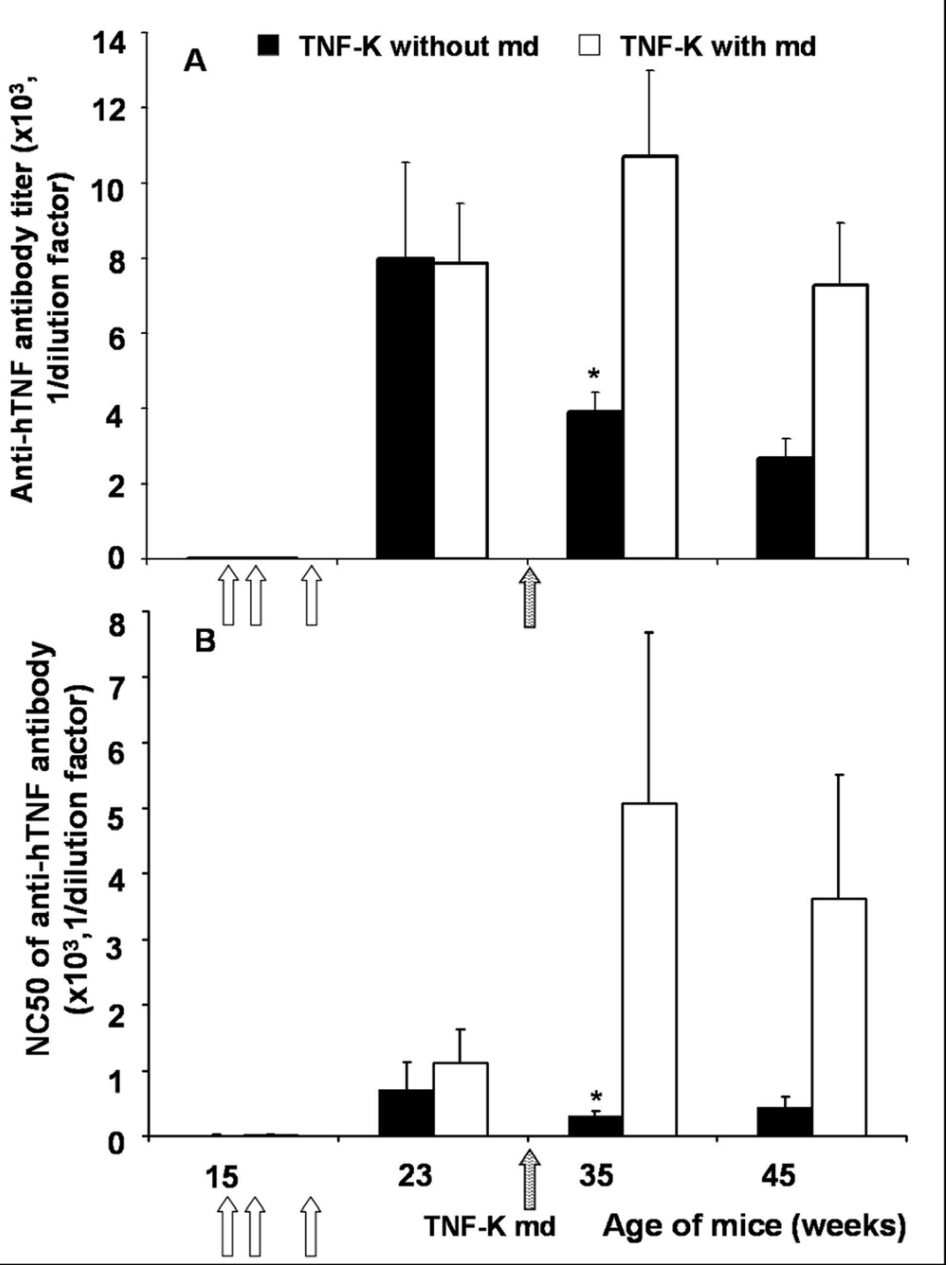
Evaluation of anti-human tumor necrosis factor-alpha (anti-hTNF-α) antibody production in TNF-α transgenic (TTg) mice immunized with TNF kinoid (TNF-K). TTg mice were immunized at 15, 16, and 19 weeks of age (open arrows) with TNF-K. **(a) **Enzyme-linked immunosorbent assay of anti-hTNF-α antibodies. **(b) **The neutralizing capacity of the anti-hTNF-α antibody was evaluated on L929 cells and is expressed as the mean of the reciprocal of the serum dilution that neutralizes 50% of hTNF-α activity (NC50). Closed histograms represent mice that did not receive the TNF-K maintenance dose (TNF-K without md) at 32 weeks of age (shaded arrow). Open histograms represent mice that did receive it (TNF-K with md). Results are expressed as mean ± standard error of the mean. *P < 0.05.

To evaluate the duration of the immune response after immunization with TNF-K in TTg mice, we assessed the titers and the neutralizing capacity of anti-hTNF-α antibodies in sera of TNF-K immunized TTg mice and of the PBS group. High levels of anti-hTNF-α antibodies were detected only in TNF-K immunized mice (Figure 4a). These antibodies were neutralizing as evaluated by L929 cytotoxic assay (Figure 4b). Mice receiving the maintenance dose at week 32 exhibited a significant increase in neutralizing anti-hTNF-α antibody titers as early as 3 weeks after the maintenance dose. Conversely, mice treated with PBS at week 32 showed a slow decrease in their neutralizing anti-hTNF-α antibody titers (Figure 4). At sacrifice, the neutralizing anti-hTNF-α antibody titers had decreased for both groups (Figure 4).

### B-memory response against TNF-α after TNF-K immunization

**Figure 5:**
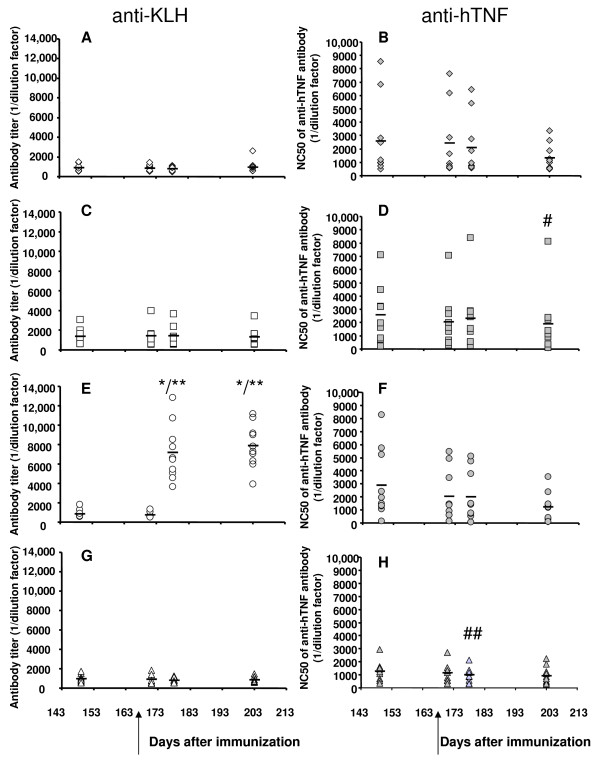
B-memory response after tumor necrosis factor kinoid (TNF-K) immunization. Thirty-six human tumor necrosis factor-alpha (hTNF-α) transgenic mice were immunized with TNF-K at 7 (day 0), 8 (day 7), and 11 (day 28) weeks of age. Bleeding was done every month from 12 weeks of age (day 38) until sacrifice. When we observed a decline of the anti-hTNF-α neutralizing antibody titer (closed symbols), we injected intraperitoneally (arrow) native hTNF-α (10 ng, n = 10, diamonds) **(a, b)**, native hTNF-α (100 ng, n = 9, squares) **(c, d)**, keyhole limpet hemocyanin (KLH) (10 μg, n = 10, circles) **(e, f)**, or phosphate-buffered saline (equivalent volume, n = 10, triangles) **(g, h)**. We studied the anti-KLH antibody titer (open symbols) and neutralizing anti-hTNF-α antibody titer (closed symbols) for 10 weeks (70 days). Each single plot represents the antibody titer of one mouse. The bold line represents the mean antibody titer at each time point. *P < 0.001 versus day 149; **P < 0.0001 versus day 171; ^#^*P *< 0.05 versus day 178; ^##^*P *< 0.05 versus day 149. NC50, mean of the reciprocal of the serum dilution that neutralizes 50% of hTNF-α activity.

We wished to evaluate the response of the immune system to native (that is, unmodified) hTNF-α after immunization with the TNF-K. We immunized TTg mice with TNF-K; once we observed a clear diminution of the neutralizing anti-hTNF-α antibody titer (Additional file 2), we injected native hTNF-α into the TNF-K immunized mice with a view to establishing whether this native hTNF-α injection induced an anti-hTNF-α response (Figure 5b, d). Control groups received injections of native KLH or PBS (Figure 5e-h). We observed that injections of native hTNF-α (10 or 100 ng) had no effect on titers of either neutralizing anti-hTNF-α antibody (Figure 5b, d) or anti-KLH antibody (Figure 5a, c). On the other hand, injections of KLH induced a dramatic increase in anti-KLH antibody titer (Figure 5e), indicating a recall response to KLH. Moreover, injection of KLH had no impact on the production of anti-hTNF-α neutralizing antibody (Figure 5f). PBS injections had no impact on the production of either anti-KLH or neutralizing anti-hTNF-α antibodies (Figure 5 g, h). Four weeks after injections by the IP route, each group of mice received IM injections of the same compound at the same dose. Anti-KLH antibody titers further increased while neutralizing anti-hTNF-α antibody titers remained stable over time (data not shown).

## Discussion

In the present study, we show in a long-term follow-up that TNF-K immunization dramatically improves the disease status of clinically established arthritis. When the active immunization was administered after the onset of active disease, its beneficial effect, mediated by the production of a high titer of neutralizing anti-hTNF-α antibodies, was evident both in clinical symptoms and in the histological indicators for arthritis. Additionally, in these experiments, we evaluated the effect of TNF-α blockade over a long-term period and showed the long-lasting efficacy and the reversible effect of TNF-K immunization. Moreover, we present evidence that no B-cell memory response to native hTNF-α was induced by TNF-K immunization.

Active immunization has previously shown its efficacy in several experimental models of human autoimmune diseases, as well as other pathologies, using cytokines cross-linked to virus-like particles of the bacteriophage Qβ [13,20,21] or complexed with KLH (kinoids) [16,22,23]. The numerous clinical trials that have been performed or that are under way support both the feasibility and the safety of the use of active immunization against self-proteins in humans [24-27].

Major questions with our active anti-cytokine immunotherapy targeting TNF-α, a pleiotropic cytokine, are the depth and the duration of the TNF-α inhibition [2]. In contrast with the previous studies, the present one has been performed with a long-term clinical follow-up (over a 36-week period). Importantly, our present data show a decrease in anti-hTNF-α neutralizing antibodies after a peak 8 weeks after immunization. At the same time, comparisons of histological scores of TNF-K-treated animals at week 27 and week 45 showed a slight progression over time of arthritides. These data support the hypotheses of both residual hTNF-α activity and the reversibility of the blockade of hTNF-α in vaccinated animals. Furthermore, a maintenance dose given 17 weeks after treatment initiation both increased the anti-hTNF-α neutralizing antibodies and ameliorated the course of disease, demonstrating that the immune system remains responsive to TNF-K immunization.

In the present study, we have also demonstrated the B-memory response to hTNF-α after TNF-K vaccination. When we stimulated the immune system of TNF-K immunized transgenic mice, we demonstrated that IP injection of KLH dramatically induced the production of new anti-KLH antibodies. This B-cell memory response to KLH was not accompanied by any increase of anti-hTNF-α neutralizing antibody titers. Furthermore, injections of native autoantigen hTNF-α after active immunization with TNF-K against hTNF-α did not induce the production of new neutralizing anti-hTNF-α autoantibodies, demonstrating no B-cell memory response to native hTNF-α. These data suggest that in physiopathological situations in which native hTNF-α production would be stimulated (for example, infections), it would not be thwarted by an immunization with TNF-K performed a long time before. Taken together, these data are consistent with the transient production and effect of neutralizing anti-hTNF-α antibodies after TNF-K immunization.

Finally, we demonstrated that TNF-K and infliximab have comparable efficacy measured by clinical parameters in our model. Moreover, once infliximab weekly injections were discontinued (at 27 weeks of age), infliximab-treated mice exhibited a worsening of arthritides over time following the withdrawal of infliximab. Histopathological scores of these animals were significantly higher than those of TNF-K immunized mice, with or without late maintenance dose.

## Conclusions

Our data show that active immunotherapy with TNF-K induced a long-lasting improvement in an RA model. The occurrence of a disease flare in previously immunized mice, the bell-shaped neutralizing anti-hTNF-α antibody curve, the increase of anti-hTNF-α neutralizing antibodies after a maintenance dose, and the absence of evidence of *in vivo *B-cell memory response to native hTNF-α are all elements supporting a favorable benefit-risk ratio for such a strategy and a transient response against hTNF-α after TNF-K immunization. Further studies should be performed to evaluate the risk of infections or tumors under TNF-K treatment in dedicated models since their occurrences are a matter of debate in patients treated with passive immunotherapies against TNF-α [28,29].

## Abbreviations

ANOVA: analysis of variance; CI: confidence interval; ELISA: enzyme-linked immunosorbent assay; hTNF-α: human tumor necrosis factor-alpha; IL: interleukin; IM: intramuscular; IP: intraperitoneal; KLH: keyhole limpet hemocyanin; mAb: monoclonal antibody; OD: optical density; PBS: phosphate-buffered saline; RA: rheumatoid arthritis; TNF-α: tumor necrosis factor-alpha; TNF-K: tumor necrosis factor kinoid; TTg: human tumor necrosis factor-alpha transgenic.

## Competing interests

GVo and ML are scientists with Neovacs SA (Paris, France), and DZ is a shareholder of Neovacs SA. TNF-K is patented and the patent is held by Neovacs SA. GVu is a scientist with Debiopharm SA (Lausanne, Switzerland). The other authors declare that they have no competing interests.

## Authors' contributions

LD and M-CB shared responsibility for the study design and manuscript preparation and helped to interpret the data and to perform the animal experiments. GVo shared responsibility for the study design and helped to interpret the data. GVu and NB shared responsibility for the study design. LS shared responsibility for manuscript preparation and helped to interpret the data and to perform the statistical analysis. DZ shared responsibility for manuscript preparation. EA helped to perform the animal experiments. ML performed the ELISA and L929 cytotoxic assay. All authors read and approved the final manuscript.

## Supplementary Material

Additional file 1 **TNF-K immunization protocol scheme**. Long-term follow -up of the experiment is represented by horizontal arrow with time expressed in week (from week 9, w9, to week 45, w45). Slashes represent discontinuation of time. A- Control group treated with PBS/ISA-51; B- TNF-K group; C- Intermittent infliximab group. The follow-up for each group (PBS, TNF-K and infliximab) is represented by a larger black line, with vertical black arrows at each time where treatment was given. IP injections, intraperitoneal injections. Click here for file

Additional file 2 **Evolution of neutralizing anti-hTNF-α antibody titers during time, in TTg mice immunized with TNF-K**. 36 TTg mice were immunized with TNFK at days 0, 7 and 28. Bleeding was done every month from day 38 post primo-injection to sacrifice. Results are expressed as mean ± SEM of all the sera of all the 36 immunized mice. Click here for file
