# Supplementary material for: Active immunization to tumor necrosis factor-α is effective in treating chronic established inflammatory disease: a long-term study in a transgenic model of arthritis
Source: Arthritis Res Ther. 2009 Dec 23;11(6):R195. doi: 10.1186/ar2897 (PMC3003505; doi:10.1186/ar2897)
Supplement: Additional file 1 — TNF-K immunization protocol scheme. Long-term follow -up of the experiment is represented by horizontal arrow with time expressed in week (from week 9, w9, to week 45, w45). Slashes represent discontinuation of time. A- Control group treated with PBS/ISA-51; B- TNF-K group; C- Intermittent infliximab group. The follow-up for each group (PBS, TNF-K and infliximab) is represented by a larger black line, with vertical black arrows at each time where treatment was given. IP injections, intraperitoneal injections. [file ar2897-S1.pdf]

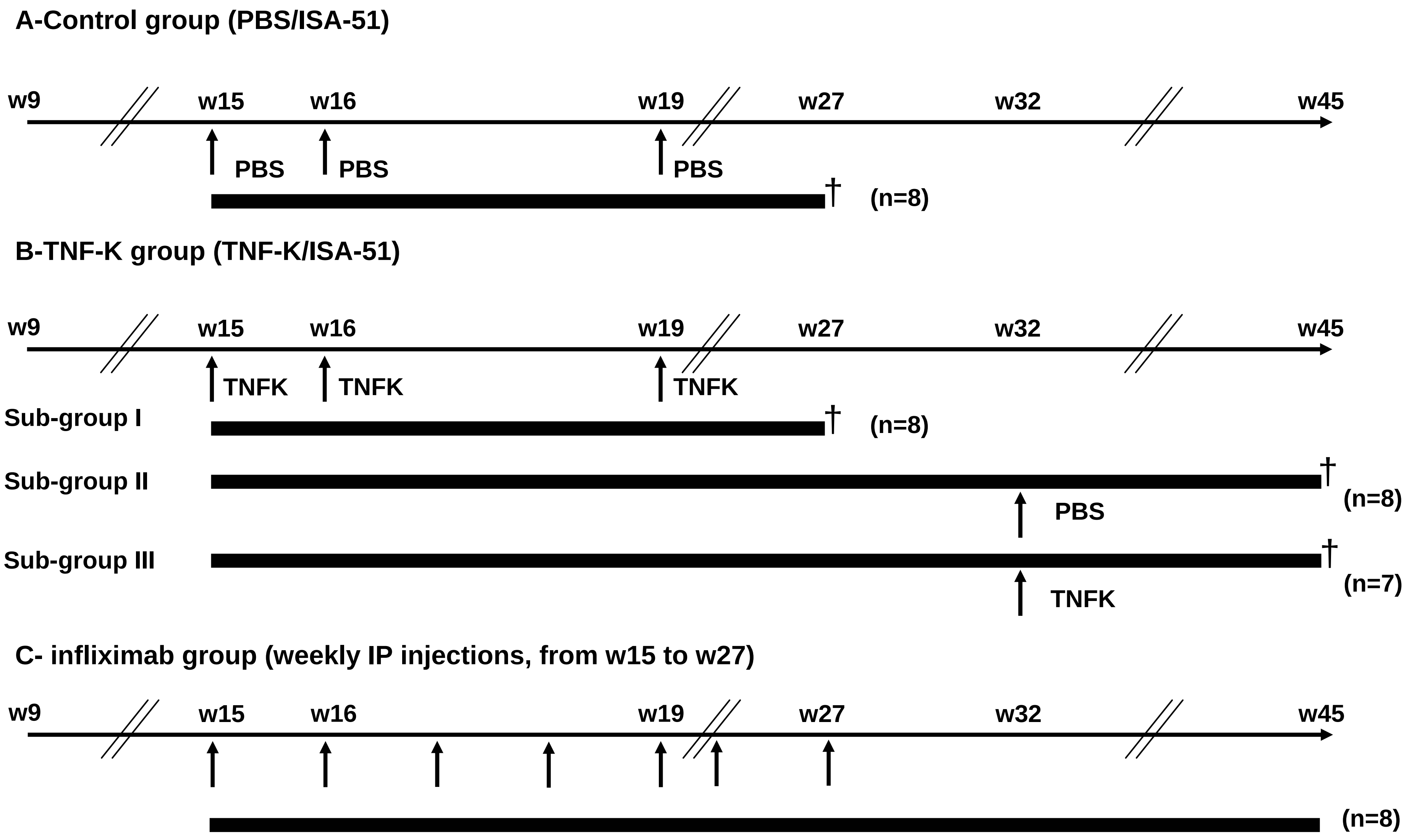

Supplemental 1- TNFK immunization protocol scheme

Long-term follow-up of the experiment is represented by horizontal arrow with time expressed in week (from week 9, w9, to week 45, w45). Slashs represent discontinuation of time. A- Control group treated with PBS/ISA-51; B- TNF-K group; C- Intermittent infliximab group. The follow-up for each group (PBS, TNF-K and infliximab) is represented by a larger black lign, with vertical black arrows at each time where treatment was given. IP injections, intraperitoneal injections.
