# Supplementary material for: Active immunization to tumor necrosis factor-α is effective in treating chronic established inflammatory disease: a long-term study in a transgenic model of arthritis
Source: Arthritis Res Ther. 2009 Dec 23;11(6):R195. doi: 10.1186/ar2897 (PMC3003505; doi:10.1186/ar2897)
Supplement: Additional file 2 — Evolution of neutralizing anti-hTNF-α antibody titers during time, in TTg mice immunized with TNF-K. 36 TTg mice were immunized with TNFK at days 0, 7 and 28. Bleeding was done every month from day 38 post primo-injection to sacrifice. Results are expressed as mean ± SEM of all the sera of all the 36 immunized mice. [file ar2897-S2.pdf]

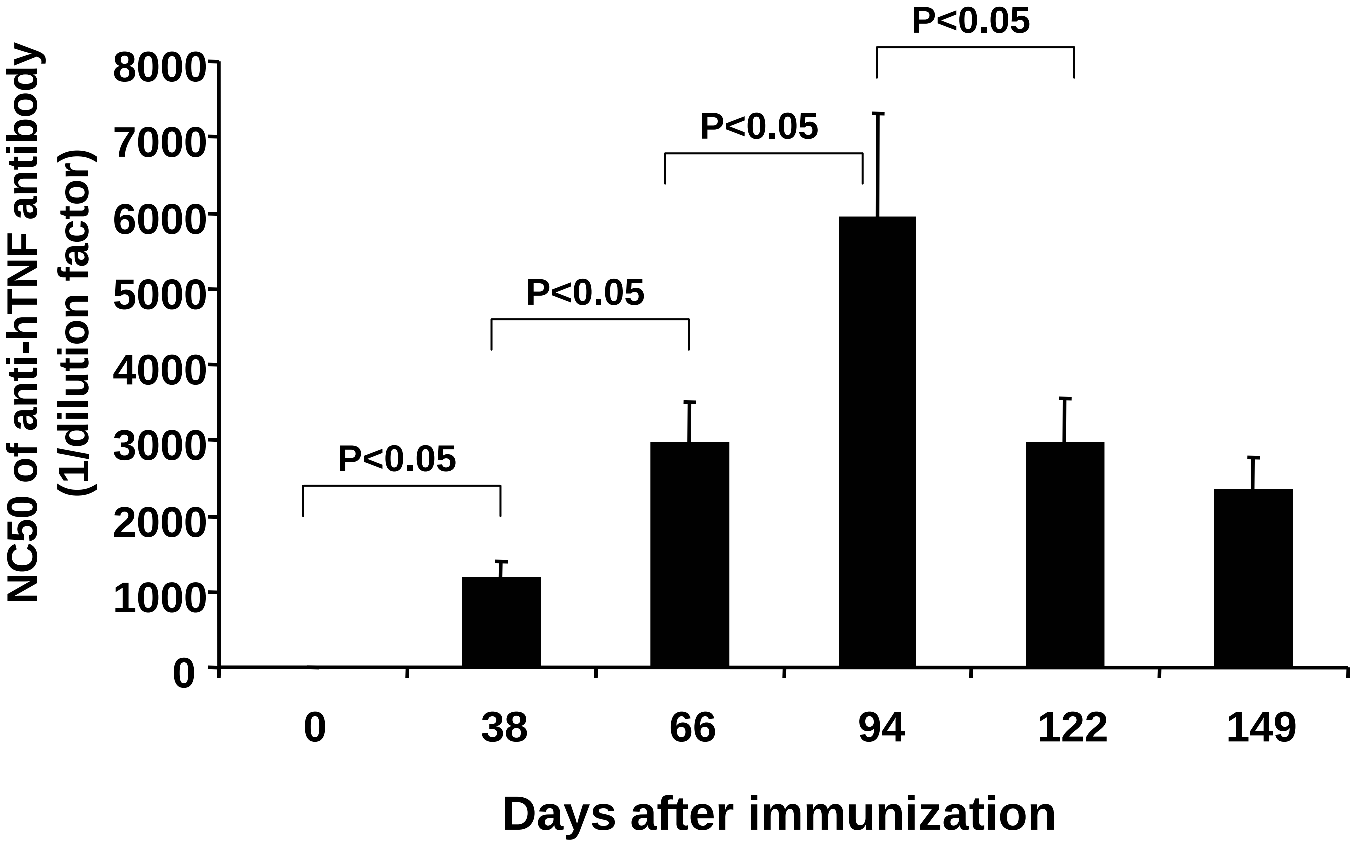

Supplemental 2- Evolution of neutralizing anti-hTNF-α antibody titers during time, in TTg mice immunized with TNFK

36 TTg mice were immunized with TNFK at days 0, 7 and 28. Bleeding was done every month from day 38 post primo-injection to sacrifice. Results are expressed as mean ± SEM of all the sera of all the 36 immunized mice.
